# Green Synthesis of Ag and Cu Nanoparticles Using *E. telmateia* Ehrh Extract: Coating, Characterization, and Bioactivity on PEEK Polymer Substrates

**DOI:** 10.3390/ma17225501

**Published:** 2024-11-11

**Authors:** Şakir Altınsoy, Kadriye Kızılbey, Hümeyra Berfin İlim

**Affiliations:** 1 Biomedical Engineering Department, Faculty of Engineering and Architecture, İstanbul Yeni Yüzyıl University, Zeytinburnu, İstanbul 34010, Türkiye; 2 Department of Natural Sciences, Faculty of Engineering and Natural Sciences, Acıbadem University, Ataşehir, İstanbul 34752, Türkiye; 3 Science and Engineering Institute, İstanbul Yeni Yüzyıl University, Zeytinburnu, İstanbul 34010, Türkiye; humeyrailim@gmail.com

**Keywords:** bioactive coatings, sustainable nanomaterials, surface modification, antimicrobial properties

## Abstract

PEEK-based implant materials have gained increasing attention as an alternative to titanium due to their biocompatibility and bone-like elasticity. However, PEEK’s surface quality and wear resistance are lower than those of metals. This study aimed to enhance the bioactivity and surface quality of PEEK by coating it with silver and copper nanoparticles synthesized via a green method using *Equisetum telmateia* Ehrh. extract. PEEK samples (Ø 25 mm, 3 mm thick) were coated with single and double layers using spray (airbrush-spray) and drop-coating methods. Comprehensive analyses including SEM, EDX, FT-IR, UV-Vis, surface roughness, release studies, antioxidant and cytotoxicity activity, and antibacterial tests were conducted on the coated samples. The results demonstrated that AgNPs and CuNPs coatings significantly improved the surface quality of PEEK. SEM analysis revealed particle sizes ranging from 48 to 160 nm for AgNPs and 50–135 nm for CuNPs, with superior dispersion obtained using the airbrush-spray method. Surface roughness measurements showed a reduction of 17–33% for AgNPs-coated samples and 7–15% for CuNPs-coated samples compared to uncoated PEEK, with airbrush-spray coatings providing smoother surfaces. Antioxidant activity tests indicated that AgNPs provided 35% higher antioxidant activity compared to CuNPs. Additionally, antibacterial tests revealed that AgNPs exhibited a higher zone of inhibition (up to 14 mm for *S. aureus* and 18 mm for *E. coli*) compared to CuNPs, which exhibited zones of 8 mm and 10 mm, respectively. This study concludes that green-synthesized AgNPs, in particular, enhance the bioactivity and surface properties of PEEK, making it a promising material for biomedical applications such as infection-resistant implants.

## 1. Introduction

Polymeric biomaterials are extensively used in fields such as tissue engineering, molecular cell biology, biodegradable material production, and controlled drug delivery [1]. Among these, thermoplastic polymers, particularly Polyetheretherketone (PEEK), have gained recognition as promising alternatives to titanium in medical and dental applications. PEEK is a semi-crystalline, linear polycyclic aromatic thermoplastic that belongs to the polyaryletherketone family. Its high biocompatibility and elastic modulus close to that of human bone make PEEK a preferred material in orthopedic, spinal, and cardiovascular implants [2].

The growing interest in PEEK for biomedical applications has led to numerous studies exploring surface modification techniques to enhance its properties. For instance, Gümüş et al. developed a PEEK-based intervertebral disc implant coated with Ti-Mg-Ag/Pt, aiming to improve antibacterial and osteoconductive properties. The Ti coating provided structural stability, while Mg and Ag contributed to bioactivity and antibacterial effects, with Pt enhancing Ag’s solubility. The modified PEEK exhibited no cytotoxic effects, making it suitable for spinal implants [3]. Similarly, Barhoumi et al. applied a TiO_2_ coating on PEEK via physical vapor deposition, significantly improving its mechanical properties and osteocompatibility [4]. In further research by Sargın et al. atmospheric plasma spray was used to coat PEEK with titanium, titanium dioxide, and hydroxyapatite to address its low bioactivity. The coatings significantly enhanced PEEK’s bioactivity, as demonstrated by in vitro testing in simulated body fluid [5]. Durham et al. employed ion-beam-assisted deposition to apply hydroxyapatite (HA) and yttria-stabilized zirconia onto cylindrical PEEK implants, resulting in superior bone regeneration and implant fixation in vivo [6]. Additionally, Liu et al. and Kratochvíl et al. focused on improving PEEK’s antibacterial properties [7,8]. Liu’s team demonstrated that silver nanoparticles (AgNPs) coatings enhanced PEEK’s antibacterial effectiveness without cytotoxicity, while Kratochvíl’s work on Cu/C nanocomposites showed strong antibacterial effects against *E. coli*, highlighting PEEK’s potential in infection-resistant implants [8].

To further enhance the properties of polymeric materials, they are often combined with metallic nanoparticles to form composites. These composites not only combine the beneficial properties of metals and polymers but also exhibit unique characteristics not found in single-phase materials [9]. Green synthesis methods, which utilize plant extracts, offer a cost-effective and eco-friendly approach to producing metallic nanoparticles such as copper, silver, and zinc [10,11,12]. These methods have become increasingly popular due to their simplicity and environmental benefits, leading to their widespread adoption in recent years. It is possible to coat the surfaces of polymers used in the biomedical field with nanoparticles using methods such as physical vapor deposition (PVD), plasma-sprayed technology, and a vacuum. A summary of scientific studies on the characterization processes applied to metal-based NPs coated on the surface of different polymeric-based substrates is reported in Table 1. One of the most important steps for the industrial development of innovative and multifunctional coatings is the development of nanometal-based surfaces.

In this study, *Equisetum telmateia* Ehrh., commonly known as great horsetail, was utilized for the green synthesis of silver and copper nanoparticles. This herbaceous perennial, widely found in moist areas across Europe, Asia, Africa, and North America, has been traditionally used to treat various health conditions. Recent research has identified bioactive compounds in *Equisetum* species, such as phenolic acids and flavonoids, which exhibit antioxidant and antimicrobial properties. These properties make them valuable as natural preservatives in the food industry, providing benefits without the adverse effects associated with synthetic additives [13].

The novelty of this study lies in the selection of PEEK for its well-established biocompatibility and suitability as an implant material, complemented by the use of green synthesis for nanoparticle production. Green synthesis is compared with conventional chemical synthesis methods, which typically require toxic chemicals and high energy consumption, making green synthesis a more environmentally friendly and sustainable alternative. By comparing the antibacterial efficacy of silver nanoparticles (AgNPs) with copper nanoparticles (CuNPs) as coating materials, the study aims to highlight the advantages of these nanotechnological approaches. The use of green synthesis not only offers an environmentally friendly and cost-effective method but also enhances the antimicrobial and antioxidant properties of the composite materials, reflecting the current relevance and innovation of this study.

The synthesized silver and copper nanoparticles were used to coat 3D-printed PEEK materials, which were produced as 25 mm diameter and 3 mm thick discs. The plant extract for green synthesis was derived from *Equisetum telmateia* Ehrh. Prior to coating, the nanoparticles were characterized using size analysis, Fourier-Transform Infrared Spectroscopy (FTIR), Scanning Electron Microscopy (SEM), and Ultraviolet–Visible (UV-Vis) spectroscopy. Two coating methods, spray and drop coating, were employed to compare the distribution and quantity of the coatings. The coated materials were further analyzed for their morphological and chemical properties using SEM-EDS, and the surface roughness was measured. The study also evaluated the antioxidant and cytotoxic activities of the composite materials and tested the antibacterial efficacy of the AgNPs and CuNPs against *S. aureus* and *E. coli* strains.

**Table 1:** Studies on the coating of various polymeric materials with metallic nanoparticles.

Ref. No	Substrate	Coating Material	Coating Method	Characterization Studies
[3]	PEEK	(Ti-Mg-Ag/Pt) Nps	Vacuum coating	Structure characterization (SEM-EDS), XRD, AFM, antibacterial activity, biocompatibility.
[4]	PEEK	TiO_2_ NPs	Physical vapor deposition (PVD)	Coating characterization (SEM-EDS), tribological analyses, Osteocompatibility test.
[8]	PEEK	AgNPs	Magnetron sputtering technology	Coating characterization (SEM-EDS), XRD, AFM, cytotoxicity, antibacterial activity.
[14]	PEEK	AgNPs	Wet chemical method	Structure characterization (SEM), thermogravimetric analysis, AFM, TEM, FTIR, antibacterial test.
[7]	PEEK	Cu/C:F Nps	Physical vapor deposition (PVD)	Structure characterization SEM, antibacterial activity.
[15]	PEEK	Air/N_2_ plasma	Low-temperature plasma	Surface characterization (SEM), XPS, antibacterial activity, biocompatibility.
[16]	PEEK	Carbon Fiber-Reinforced (CFR)	Injection	Surface roughness, structure characterization (SEM-EDS), in vitro cytocompatibility evaluation, antibacterial test.
[17]	PEEK	Pure Ti	Electron beam deposition	Biological properties (in vitro tests and in vivo tests), SEM-EDS, XRD, TEM.
[18]	PEEK	N_2_ plasma	Plasma technology	Surface characterization (SEM), AFM, XPS, antibacterial activity, biocompatibility.
[19]	PEEK	Porous PEEK and Pure Ti	Plasma-sprayed technology	Structure characterization (SEM-EDS), biological properties (in vitro tests and in vivo tests).
In this study	PEEK	AgNPs-CuNPs	Airbrush spray and drop-casting	ZetaSizer, UV–Vis, FT-IR, SEM-EDX, surface roughness, release, antioxidant, cytotoxicity, antibacterial activity.

## 2. Materials and Methods

### 2.1. PEEK Material

For this study, samples made of Polyetheretherketone (PEEK) material with dimensions of Ø 25 × 3 mm were produced. For the production of the PEEK materials used in this study, services were provided by AddPark-Advanced Manufacturing and Technology Centre. The three-dimensional design of the PEEK samples used in the experiments was created using computer-aided design software (V.58.0.0 AutoCAD 2025) and transferred to a 3D printer. The samples were produced using a material called medical PEEK with the Medical 3D Printing-Apium M220 device.

### 2.2. Preparation of Equisetum Telmateia Ehrh. Extract

In this study, the plant *Equisetum telmateia* Ehrh. was used to synthesize metal nanoparticles through a green synthesis method. The dried *E. telmateia* Ehrh. was ground into a powder using a blender, and 20 g of it was weighed. The weighed plant material was placed into a 600 mL beaker, and 300 mL of distilled water was added. The plant–water mixture was left to steep in a magnetic stirrer at 70–80 °C for 4 h without boiling. After the mixture was allowed to cool to room temperature, it was filtered using filter paper. The obtained extract was then stored in the refrigerator at 4 °C for future use [20].

### 2.3. Preparation of AgNPs and CuNPs

The production of metal nanoparticles involves quite expensive and labor-intensive methods. In addition to these challenges, producing metal nanoparticles through the green synthesis method is both an economical and eco-friendly approach. Furthermore, its ease of applicability makes green synthesis highly preferable [11]. Plant extracts act as natural reducing agents in nanoparticle synthesis. Polyphenols and flavonoids in plant extracts reduce metal ions (e.g., Ag⁺ and Cu^2^⁺) and convert them into metal nanoparticles. This process plays a critical role in the formation of nanoparticles. Compounds such as polyphenols convert metal ions into stable nanoparticles and also stabilize these nanoparticles by forming a coating around them. Once the nanoparticles are formed, organic compounds in plant extracts such as flavonoids and polyphenols adhere to the surface of the nanoparticles and this partially prevents the particles from aggregating. These compounds act as a coating that stabilizes the nanoparticles. Thus, the particles become more stable and can remain in a solution without sticking together. In the classical synthesis method, stabilizers are used for this process [21]. After the reduction process, the metal ions form nucleation centers for the growth of the nanoparticles. In the nucleation stage, reduced metal atoms come together to form nanoparticle nuclei. These nuclei then grow into larger nanoparticles. The nucleation and growth stages are critical in determining particle size and morphology [22]. The visible changes in solution color during the synthesis of metal nanoparticles are related to a phenomenon called surface plasmon resonance. Metal nanoparticles have a plasmonic effect that causes free electrons to vibrate on the metal surface. These vibrations absorb certain wavelengths of light and scatter the rest. Different nanoparticle sizes and shapes cause different wavelengths of light to be absorbed when interacting with this light, which results in a change in solution color [23]. For the synthesis of AgNPs, 4 g of silver nitrate (AgNO_3_) salt was weighed and mixed with 20 mL of distilled water to prepare an AgNO_3_ solution. The entire prepared AgNO_3_ solution was added to the *E. telmateia* Ehrh. extract at room temperature in an ultrasonic bath and with a mechanical stirrer (400 rpm) using an automatic pipette. A color change was observed as the AgNO_3_ solution was added to the extract, and by the end of the process, the extract–solution mixture turned a blackish color. After the mixing process, NaOH was added to the extract–AgNO_3_ mixture, raising the pH from 5.1 to 11. The mixture was then centrifuged, and the liquid portion was discarded. The remaining precipitate was washed three times: twice with water and once with ethanol. The obtained AgNPs were left to dry for 2 days at 50 °C in a WiseVen brand oven. The dried AgNPs were stored in a desiccator for coating.

For the synthesis of CuNPs, E. telmateia Ehrh. was used, similar to the synthesis of AgNPs. Copper sulfate (CuSO_4_) salt was weighed at 250 mg and dissolved in 10 mL of distilled water. The entire CuSO_4_ solution was added to the plant extract using a pipette in a dark room at 60 °C with a magnetic stirrer at 800 rpm, and the mixture was left to stir for 2 h. After mixing, the pH was adjusted from 3.8 to 9 using NaOH. The CuNPs were centrifuged, and the solid phase underwent a washing process which was repeated three times: twice with distilled water and once with ethanol. The washed CuNPs were dried overnight at 50 °C in an oven. The dried CuNPs were stored in a desiccator.

### 2.4. Coating of PEEK with Nanoparticles

The synthesized AgNPs and CuNPs organic nanostructures were coated onto the PEEK substrate using two different coating methods: airbrush-spray and drop-casting. Before the coating process began, in order to obtain cleaner surfaces, the PEEK discs were kept in ethanol in P select, an Ultrasons HD model ultrasonic bath device, for 15 min and then dried in an oven at 75 °C for 15 min. For the drop-casting method, AgNPs that could coat the surface of the PEEK samples were weighed at 10 mg and mixed with 10 mL of distilled water. Using an automatic pipette, 0.5 mL of the liquid was applied to each layer in three drops on the cleaned PEEK samples. The coated substrate specimens were subjected to a drying operation for 1 h at 60 °C using a WiseVen brand oven device. The dryness of the samples was checked at regular intervals to prevent cracks from forming on the coating’s surface. The coating operation was repeated under the same conditions for the double-coated specimens. In the drop-casting method for CuNPs, the CuNPs stored in liquid form were stirred in a magnetic stirrer for 10 min to mitigate the risk of agglomeration. The coating steps for CuNPs were repeated in the same manner as for AgNPs.

**Figure 1:**
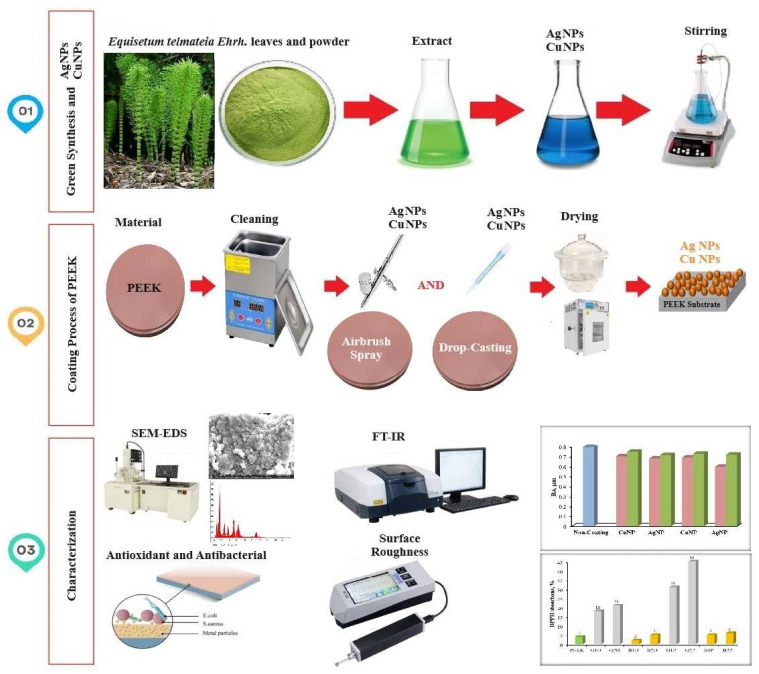
The schematic view of the experimental steps.

An air compressor (HSENG-AS186) was used for the airbrush-spray coating method. Simultaneously to the drop-casting method, AgNPs that would coat the surface of the PEEK samples were weighed at 10 mg and mixed with 10 mL of distilled water. First, 1 mL of the AgNPs solution was collected with an automatic pipette and placed into the liquid chamber of the air compressor. The spray nozzle of the air compressor was positioned vertically 70 mm above the cleaned PEEK samples, with the pressure set at 3 atm. Spraying was carried out for 5 s, with a 10 s wait between each spray, until the liquid in the chamber was exhausted. Each layer was allowed to dry in an oven at 60 °C for 1 h, with the samples checked periodically to prevent cracking. The coating operation was repeated under the same conditions for the double-coated specimens. The steps used for the airbrush-spray coating of AgNPs were also repeated for the coating of CuNPs. Five sample coatings were applied for each coating parameter. The experimental coating details are shown in Table 2. The schematic representation of the experimental steps is illustrated in Figure 1.

### 2.5. Characterization

#### 2.5.1. Nanoparticles Characterization

AgNPs and CuNPs were synthesized from *E. telmateia* Ehrh using the green synthesis method, and their particle size (Z-average) was determined via dynamic light scattering using a Malvern Zetasizer Nano device (Malvern Panalytical, Worcestershire, UK). Measurements were performed with three repetitions at 25 ± 0.1 °C using 0.8872 cP viscosity and 1.330 refractive index values for solutions. In the dynamic light scattering (DLS) method, the sizes of particles exposed to Brownian motion with a laser beam are determined using the Stokes–Einstein equation [24]. The polydispersity Index (PDI) is a measure of the breadth of the particle size distribution in a solution. PDI is calculated from correlation function data obtained by dynamic light scattering (DLS) and is usually calculated automatically by DLS analyzers. The UV–Vis absorption spectrum of these nanoparticles was analyzed by scanning across a wavelength range of 200–800 nm at room temperature with a Shimadzu UV mini-1240 spectrophotometer (Kyoto, Japan). FT-IR spectroscopy (IRAffinity-1, Shimadzu, Kyoto, Japan), which measures the absorption of electromagnetic radiation in the midinfrared region (4000–400 cm^−1^) [25], was also employed to characterize the samples. For SEM-EDS analysis, Thermo Fisher Scientific Quattro S (Waltham, MA, USA) and Zeiss/Evo 10 (Oberkochen, Germany) SEMs were utilized to investigate the morphology of surface-modified PEEK samples [26]. The surface roughness values of both non-coated and AgNPs/CuNPs coated PEEK specimens were measured using a Mitutoyo Surftest 210 surface profilometer (Mitutoyo, Japan). In this study, surface roughness measurements were taken from five different points on each sample coated using drop-casting and airbrush-spray methods, and the averages of these measurements were calculated. Thus, surface roughness across different coating thicknesses was evaluated.

#### 2.5.2. Release Test

Release studies of the coated samples were conducted in an in vitro environment using a phosphate-buffered saline (PBS) solution at pH 7.4, at 37 °C, and 60 rpm in a refrigerated shaking incubator (Mikrotest-MCS-55, İstanbul, Türkiye). The samples coated with AgNPs and CuNPs were placed into beakers containing 3 mL of PBS solution. At the 1st h, 2nd h, 3rd h, 4th h, 1st day, 2nd day, 3rd day, 4th day, and 7th day, 0.5 mL was drawn from the media containing the samples, and the same amount of fresh PBS solution was added. The analysis of the collected liquids was performed using a UV-Vis Spectrophotometer (Shimadzu UVmini-1240, Kyoto, Japan) [27].

#### 2.5.3. Antioxidant Activity Test

The antioxidant potential of our samples was tested using the 2,2-diphenyl-1-picrylhydrazyle (DPPH) free radical scavenging method [28]. Each sample was immersed in 2 mL of 1 mL of a 1:1 (*v*/*v*) mixture of TRIS buffer at pH 7.4 and DPPH (100 μM in ethanol). Samples were incubated for 20 min in the dark and the absorbance of the solution was recorded at 517 nm. DPPH absorbance reduction was calculated by the following equation: Reduction% = [1 − (A_sample_/A_DPPH_)] × 100, with A_sample_ representing the absorbance after 20 min in the dark and A_DPPH_ representing the absorbance of DPPH solution before the addition of the solids.

#### 2.5.4. In Vitro Cytotoxicity Analysis

Thiazolyl Blue Tetrazolium Bromide (MTT) assay is a colorimetric cytotoxicity assay based on measuring the metabolic activity levels of cell mitochondria. The principle of the MTT method is based on the mitochondrial dehydrogenase enzyme found in the mitochondria of living cells, which oxidizes lactate to pyruvate, while the NADH^+^ that is released turns the yellow MTT reactant into purple formazan crystals. The formation of purple formazan crystals is directly proportional to active metabolism and cell viability [29]. In this study, the hydrophilic, yellow-colored formazan salt MTT (3-(4,5-dimethylthiazol-2-yl)-2,5-diphenyltetrazolium bromide) was preferred for assessing cell metabolic activity. In this test, the increase in cell proliferation enhances mitochondrial dehydrogenase enzyme activity and then this enzyme converts the MTT salt into apolar, purple-colored MTT formazan crystals. These crystals become soluble in DMSO or isopropyl alcohol as a result of this transformation. The dye intensity obtained from the MTT assay is directly related to the cell number [30].

L929 mouse fibroblast cells were used as the test cells, and the biocompatibility performance of the coated PEEK samples was evaluated according to the ISO-10993-5 standard [31]. Nine samples, including both coated and non-coated PEEK, were placed in wells containing 5 mL of culture medium. At specific time intervals, on day 1 and day 4, 0.2 mL of liquid was taken from each well. MTT tests were performed on the collected liquids, and the absorbance values were analyzed using a spectrophotometer as part of the ELISA method.

#### 2.5.5. Antibacterial Activity Test

To investigate the antibacterial performance of PEEK samples coated with AgNPs and CuNPs, tests were conducted using *E. coli* and *S. aureus* bacteria. The bacterial activity was determined using the *agar well* diffusion method. Bacterial strains were incubated overnight at 37 °C and 180 rpm, spread onto agar plates, and wells with a diameter of 8 mm were made in the 4 mm thick agar (ensuring a uniform diameter for each well using the reverse side of a pipette tip). For each bacterial strain, Ag and Cu nanoparticles were dissolved in distilled water at concentrations of 0.5 mg/mL and 1.0 mg/mL and exposed to UV light for 15 min. The prepared solutions were added to the wells in the agar, and the plates were incubated at 37 °C for 24 h. After incubation, the zones of inhibition around the wells were measured to evaluate bacterial activity [32].

## 3. Results and Discussion

During the synthesis, silver nanoparticles were obtained with 71% yield, while copper nanoparticles were obtained with 65.25% yield. Considering that the plant extract used is the same, it is thought that the difference in yield is due to the different properties of the metals. Even using the same plant extract, copper nanoparticles can be more difficult to synthesize than silver. Copper reduction and stabilization often require more precise pH and temperature conditions. At lower temperatures or inappropriate pH levels, copper nanoparticles may be less efficient. Silver nanoparticles have more stable physical and chemical properties. This stability leads to more efficient reduction and stabilization of silver ions. Silver is known for its high electrical conductivity and resistance to oxidation, which increases the synthesis efficiency [33]. Copper is a metal that can oxidize much faster than silver. This can cause a loss of efficiency due to oxidation during the synthesis of copper nanoparticles. The oxidation process affects the stability of the nanoparticles, and therefore the yield of copper nanoparticles is generally lower [34].

### 3.1. ZetaSizer Particle Size Analysis Results

**Figure 2:**
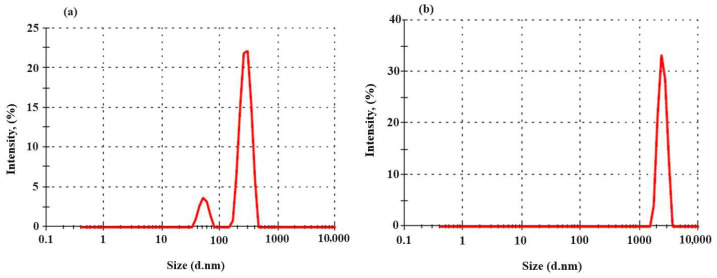
Size distribution of AgNPs (**a**) and CuNPs (**b**) by intensity.

The particle size analysis of nanoparticles synthesized using the green synthesis method from *E. telmateia* Ehrh. was repeated three times, and the average value was used. The particle size analysis for AgNPs, as shown in Figure 2a, indicated an average size of 483 nm with a polydispersity indexes value (PDI) of 0.408. Similarly, Figure 2b presents the analysis of CuNPs synthesized through green methods, revealing a particle size of 2341 nm and a PDI value of 0.291. These results reflect the respective size distributions and heterogeneity of both nanoparticle samples.

The Zeta potential of silver nanoparticles was measured in −24 mV and copper nanoparticles as −11 mV. The zeta potential for silver nanoparticles produced by green synthesis generally varies between −20 mV and −40 mV. The zeta potential of AgNPs generally shows higher stability. This is because silver nanoparticles are determined to be better stabilized by bioactive compounds such as polyphenols and flavonoids in plant extracts. If the zeta potential is around −20 mV, this indicates that the silver nanoparticles have reasonable stability, but when the stability is −30 mV or higher, this is indicative of a much more stable system. These particles remain more homogeneous in solution and have a reduced tendency to agglomerate [35]. The zeta potential of copper nanoparticles is usually between −10 mV and −40 mV. However, since CuNPs are more susceptible to oxidation, the zeta potential can sometimes be lower, which can negatively affect the stability of the nanoparticles. Zeta potential values below −10 mV indicate that the stability of the colloidal system is poor and the nanoparticles can easily aggregate in solution. A low zeta potential indicates that the electrostatic repulsion forces between the nanoparticles are insufficient, and therefore the particles tend to aggregate [36].

A review summarizing several green synthesis methods reported that the PDI of AgNPs commonly falls between 0.2 and 0.4, depending on the plant extract and synthesis conditions. For instance, nanoparticles synthesized with Azadirachta indica (Neem) extract showed a PDI of around 0.3, reflecting good size distribution and stability [37]. A study on silver nanoparticles synthesized using black mulberry (*Morus nigra*) extract found that the AgNPs had a particle size of 170.17 nm with a PDI of 0.281. This low PDI indicates a relatively homogeneous particle size distribution, which suggests the synthesis method produces stable nanoparticles with minimal aggregation. Additionally, these AgNPs exhibited significant antioxidant, antibacterial, and anti-inflammatory activities, enhancing their potential for biomedical applications [21]. Another study used Salvia officinalis extract for the green synthesis of AgNPs. The resulting particles had a size range of 10–50 nm, with a PDI of 0.324. This indicates a slightly wider size distribution compared to the black mulberry study but is still within the acceptable range for nanoparticle stability. The authors highlight that the use of natural extracts not only provides eco-friendly synthesis but also effectively stabilizes the nanoparticles [34]. These studies show that PDI values around 0.2 to 0.4 are typical for AgNPs synthesized through green methods, reflecting reasonable to good stability and homogeneity in size distribution. Comparing our results to these, a PDI of 0.408 for our AgNPs is slightly higher but still within a reasonable range, indicating that further optimization could enhance the stability of the nanoparticles.

A study on copper nanoparticles synthesized using *Jatropha curcas* leaf extract found that the CuNPs had an average size of 10 ± 1 nm with a PDI of 0.245. This relatively low PDI indicates a narrow size distribution, suggesting that the plant extract acted as an effective stabilizing agent, preventing agglomeration and leading to well-dispersed nanoparticles. The study highlights the role of flavonoids, tannins, and glycosides in the synthesis and stabilization of the nanoparticles [38]. Another study utilized *Ocimum sanctum* leaf extract for the synthesis of CuNPs. The particles were found to have an average size of 11 nm, and the PDI value was reported as 0.301, indicating a slightly broader distribution but still within a range considered stable. The phytochemicals present in the extract, such as alkaloids and flavonoids, were found to contribute to both the reduction of copper ions and the stabilization of the nanoparticles [39]. A different study focused on the antifungal properties of CuNPs synthesized using plant extracts reported a PDI of 0.298 for nanoparticles with an average size of 15 nm. The PDI value indicates good dispersion, and the study further demonstrated the efficacy of these particles as eco-friendly antifungal agents, supporting the use of green-synthesized CuNPs in agriculture [40]. Given these studies, it is shown that the PDI values of copper nanoparticles (CuNPs) obtained by green synthesis are generally between 0.2 and 0.3. These values reflect stable and well-dispersed nanoparticle formations. This suggests that the plant extracts used during the synthesis effectively stabilize the nanoparticles and limit agglomeration.

### 3.2. UV-Vis Spectroscopy Analysis Results

**Figure 3:**
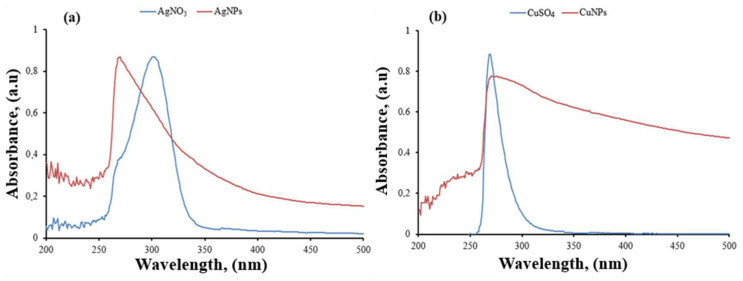
UV-Vis spectrum of AgNO_3_ and AgNPs (**a**), and UV-Vis spectrum of CuSO_4_ and CuNPs (**b**).

UV-Vis spectroscopy was conducted to investigate the surface plasmon resonance (SPR) of the synthesized nanoparticles within the organic biomatrix. SPR involves the absorption of visible electromagnetic energy due to the collective oscillation of electron transfer occurring on the surface of the nanoparticles [41]. In this study, AgNPs and CuNPs were synthesized using a green synthesis method with AgNO_3_ and CuSO_4_ solutions. The UV-Vis spectrum results for AgNO_3_, AgNPs, CuSO_4_ and CuNPs are shown in Figure 3a,b, respectively.

In the graph shown in Figure 3a, the AgNO_3_ solution exhibits a peak at 305 nm, while in the same graph, the peak for AgNPs shifts to a wavelength of 271 nm, indicating a different characteristic peak. Based on these results, it is believed that AgNPs have formed from the silver nitrate solution. Other analyses support this formation. In the graph presented in Figure 3b, the CuSO_4_ solution shows a peak at 270 nm, while the peak for CuNPs shifts to a wavelength of 278 nm, indicating a different characteristic peak. Based on these results, it is believed that CuNPs have formed from the copper sulfate solution. Other analyses also support these conclusions [42].

### 3.3. FT-IR Analysis Results

**Figure 4:**
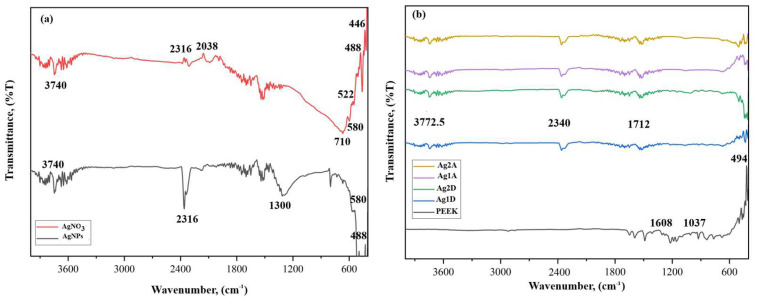
FT-IR spectrum AgNO_3_ and AgNPs (**a**), and PEEK samples coated with AgNPs (**b**).

The structural characterizations of the obtained AgNPs, CuNPs, coated PEEK samples, and the *Equisetum telmateia* Ehrh. plant were performed using FT-IR spectroscopy. Figure 4a,b show the FT-IR spectrum results of the comparative AgNO_3_-AgNPs and the samples coated with AgNPs, respectively.

When examining the graphs given in Figure 4a, peaks were observed at 3740 cm^−1^, 2316 cm^−1^, 2038 cm^−1^, 710 cm^−1^, 580 cm^−1^, 522 cm^−1^, 488 cm^−1^, and 446 cm^−1^ bands in the FT-IR spectrum of AgNPs. Small shifts and changes in the wave numbers between 400 cm^−1^ and 2000 cm^−1^ in the FT-IR spectra of AgNPs obtained with reducing materials confirm the interaction of functional groups with AgNPs. The band in the 3740 cm^−1^ range, known as the X-H region, originates from the stretching vibrations of phenol and carboxylic groups, as well as the stretching vibrations of N-H and -OH groups [43]. The peaks between 2000 and 2300 cm^−1^ in the AgNPs spectrum are thought to result from the interaction of silver with molecules present in the plant extract [44]. The 2316 cm^−1^ and 2038 cm^−1^ bands represent C-H stretching. Other bands in the range of 446–710 cm^−1^ show a spectrum consisting of C=C out-of-plane bending peaks. The inclusion of silver salt into the plant extract under optimal conditions leads to the infiltration of reducing agents in the plant extract, thereby enabling the formation of stable silver nanoparticles and the encapsulation of silver ions [45]. Considering the plant extract used in this study, it can be suggested that alkynes, sulfur compounds, alcohol and phenolic compounds, proteins, and other water-soluble biomolecules act as reducing and stabilizing agents [46,47]. Thus, proteins will likely form a coating over the metal nanoparticles (coating of silver nanoparticles), preventing the aggregation of the particles and stabilizing them in the medium. This indicates that biological molecules can function in the formation and stabilization of silver nanoparticles in aqueous environments [48].

Upon examining the FT-IR spectrum of non-coated PEEK and PEEK samples coated with AgNPs shown in Figure 4b, peaks were observed in the FT-IR spectrum graphs of PEEK materials coated with single or double layers of AgNPs using airbrush-spray and drop-casting methods, at bands of 3772.5 cm^−1^, 2340 cm^−1^, 1712 cm^−1^, 1608 cm^−1^, 1037 cm^−1^, and 494 cm^−1^. The band in the 3772.5 cm^−1^ range belongs to X-H stretching bands. The 1712 cm^−1^, 1608 cm^−1^, and 1037 cm^−1^ bands correspond to the stretching vibrations of the C=O, C=C, and C-O carbonyl groups, respectively [49]. The 494 cm^−1^ band shows a spectrum consisting of out-of-plane bending peaks of C=C [50].

**Figure 5:**
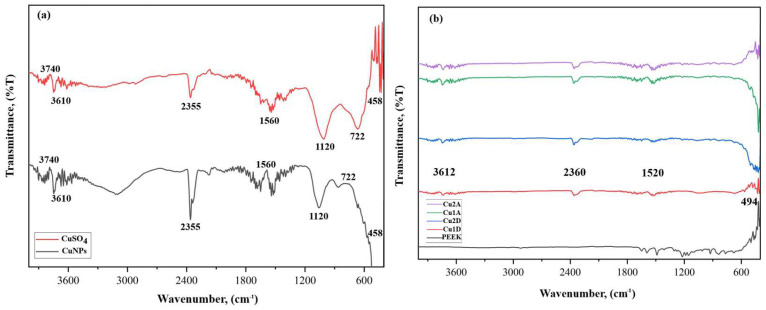
FT-IR spectrum CuSO_4_, CuNPs (**a**), and PEEK samples coated with CuNPs (**b**).

In this study, the structural composition of CuNPs was also determined using FT-IR spectrum analysis. In Figure 5a,b shows the FT-IR spectrum results of the comparative CuSO_4_-CuNPs and the samples coated with CuNPs, respectively.

When examining the graphs given in Figure 5a, peaks were identified in the FT-IR spectrum of CuNPs at bands of 3740 cm^−1^, 3610 cm^−1^, 2355 cm^−1^, 1560 cm^−1^, 1120 cm^−1^, 722 cm^−1^, and 458 cm^−1^. The bands in the 3740 cm^−1^ and 3610 cm^−1^ ranges, known as the X-H region, arise from the stretching vibrations of phenol and carboxylic groups, as well as N-H and -OH groups [51]. The study involves a green synthesis method using plant extract. Therefore, plant-derived structures can be observed on the nanoparticle surface. Peaks at 2355 cm^−1^, 1560 cm^−1^, and 1120 cm^−1^ in the CuNPs spectrum correspond to O-H stretching, C=N stretching, and C-O stretching, respectively. The peaks at 722 cm^−1^ and 458 cm^−1^ represent aromatic C-H stretching [51,52]. The characteristic peak observed at 597 cm^−1^ represents the Cu-O stretching vibration and confirms the formation of CuNPs [42].

The FT-IR spectra of non-coated PEEK and CuNPs-coated PEEK samples are shown in Figure 5b. The peaks at bands of 3612 cm^−1^, 2360 cm^−1^, and 1520 cm^−1^ are observed in the FT-IR spectrum graphs of PEEK materials coated with single or double layers of CuNPs using airbrush-spray and drop-casting methods. The band in the 3612 cm^−1^ range corresponds to X-H stretching bands. The 2360 cm^−1^ and 1520 cm^−1^ bands correspond to the stretching vibrations of O-H and C=N groups, respectively. Other lower peaks correspond to a spectrum consisting of out-of-plane bending peaks at 494 cm^−1^ [51,52].

### 3.4. SEM-EDS Analysis Results

#### 3.4.1. SEM Analysis

**Figure 6:**
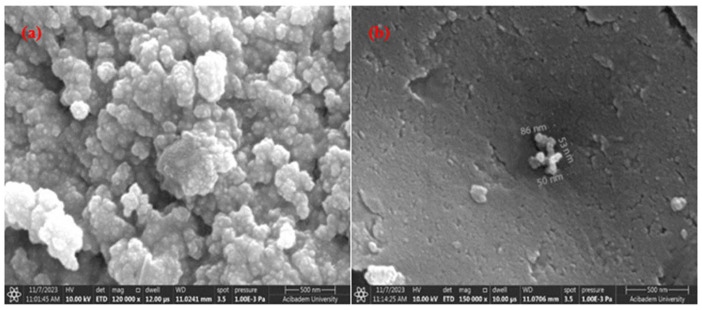
SEM images of AgNPs (**a**) and CuNPs (**b**).

In this study, SEM analyses were performed to detect the particle distribution and size of the pure *Equisetum telmateia* Ehrh. extract-based AgNPs and CuNPs. Additionally, SEM analyses were performed for surface examinations of PEEK samples coated with AgNPs and CuNPs using spraying and drop-casting methods under different parameters. Figure 6a,b show the SEM images of AgNPs and CuNPs synthesized using *Equisetum telmateia* Ehrh. extract.

When examining the SEM images of AgNPs shown in Figure 6a, it is clearly seen that most of the silver particles are spherical in shape, and large agglomerations are not present. It is understood that the nanoparticles depicted and measured in the figure have particle sizes ranging from 53 to 114 nm and are well dispersed.

When the SEM images of CuNPs shown in Figure 6b are examined, it is observed that the particles are connected in certain areas, forming peaks, and this structure appears to be amorphous (agglomerated). From the SEM images, it is understood that the particles have different diameters and sizes. Agglomeration is a common problem encountered in nanoparticle synthesis [24]. It is seen that the particles depicted and measured in the figure have particle sizes ranging from 50 to 56 nm and are well dispersed.

**Figure 7:**
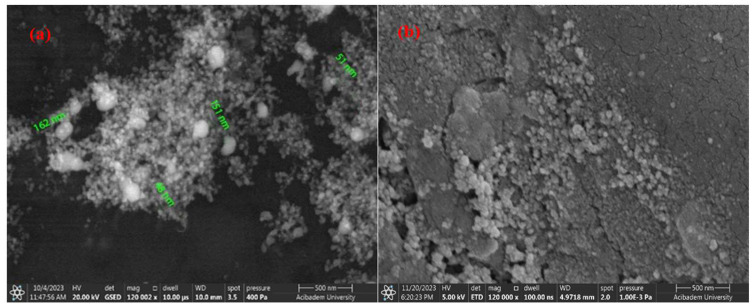
SEM images of AgNPs using the drop-casting method: (**a**) single-layer coating; (**b**) double-layer coating.

**Figure 8:**
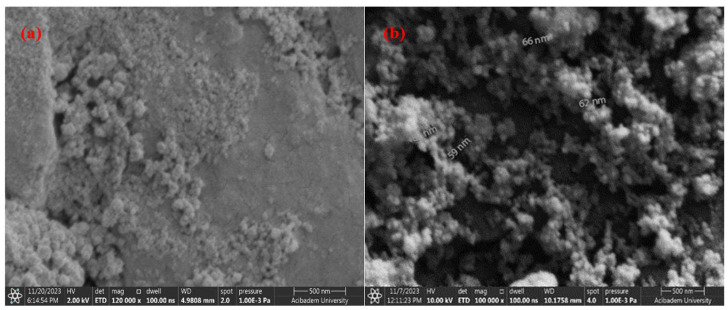
SEM images of AgNPs using the airbrush-spray method: (**a**) single-layer coating; (**b**) double-layer coating.

Figure 7a,b and Figure 8a,b, respectively, show SEM images of the coated surfaces of PEEK samples covered with AgNPs using drop-casting and airbrush-spray methods. Drop-casting is a simple method in which a nanoparticle solution is spread over a surface and left to dry. However, this process can sometimes lead to uneven particle distribution because as the liquid evaporates, particles can group together, causing clumps (agglomeration). This happens because the liquid does not always spread evenly, leading to thicker areas and less uniformity. While it is easy to use, drop-casting can be tricky for perfectly smooth and even coatings. Adjustments, like using the right solvent and controlling drying conditions, can help improve the results [53,54]. Spray-coating, on the other hand, allows for a more controlled and even spread of particles. The nanoparticle solution is sprayed in a fine mist, which helps distribute the particles more uniformly over a larger area. This method is great because it reduces the chance of clumping and provides a smoother coating. By adjusting factors like the distance between the spray nozzle and the surface or the speed of the spray, you can ensure the particles settle evenly. Additionally, when heated properly, the solution dries quickly, helping prevent particles from forming large clusters [55,56].

Figure 7 and Figure 8 show that the surfaces coated with both single and double layers using the two different coating methods on PEEK materials are successfully coated. SEM images of AgNPs clearly indicate that the average size of the nanoparticles is in the nanometer range. It has been distinctly observed that with the increase in AgNPs concentration in double-layer coatings using the drop-casting method, the particle size changes. Figure 7a,b show that the particles have sizes ranging from 48 to 160 nm, while Figure 8a,b show that the particles range from 59 to 80 nm.

Examining the surface morphologies of both coating methods, it has been determined that the coatings are acceptably rough, crack-free, and single-layered. With the increase in coating thickness using the drop-casting method, a noticeable increase in agglomeration between particles has been observed. In contrast, when coating is carried out using the airbrush-spray method, the particles are spherical in shape and agglomeration is reduced. In spray techniques, nanoparticles slow down due to friction as they pass through the liquid and accumulate on the material surface. The effects of air pressure spraying methods on coating surface morphologies include covering a wider area on the surface and ensuring that the accumulated particle structure is cleaner [57,58]. Overall, it can be said that AgNPs are smaller in size, spherical in shape, and spread more evenly and densely throughout the coating layer compared to CuNPs.

**Figure 9:**
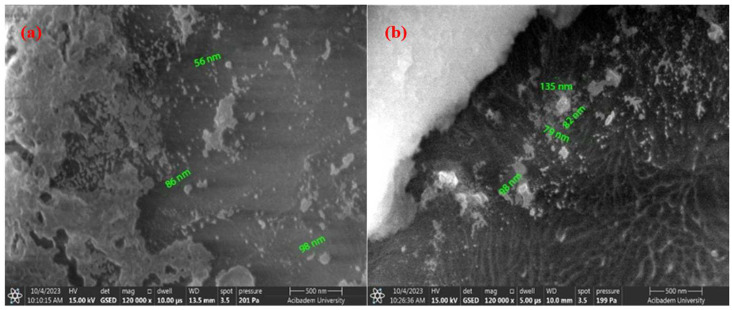
SEM images of CuNPs using the drop-casting method: (**a**) single-layer coating; (**b**) double-layer coating.

**Figure 10:**
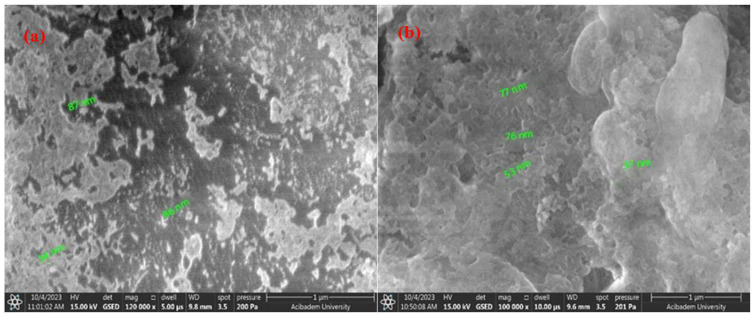
SEM images of CuNPs using the airbrush-spray method: (**a**) single-layer coating; (**b**) double-layer coating.

Figure 9a,b and Figure 10a,b, respectively, show SEM images of the coated surfaces of PEEK samples covered with CuNPs using drop-casting and airbrush-spray methods.

Figure 9 and Figure 10 show that the surfaces coated with both single and double layers using the two different coating methods for PEEK materials are successfully coated. It is important to examine both methods to enhance homogeneity. SEM images of CuNPs clearly indicate that the average size of the nanoparticles is in the nanometer range. It has been observed that the particle size does not change significantly with the increase in Cu concentration in double-layer coatings made using the drop-casting method. Figure 9a,b show that the particles have sizes ranging from 56 to 135 nm, while Figure 10a,b show that the particles range from 37 to 87 nm.

Compared to the airbrush-spray method, the coating resulting from the drop-casting method shows an uneven surface morphology on the PEEK surface and is not distributed homogeneously. However, it is observed that coatings with both methods are crack-free and single-layered. In the drop-casting method, the particles are observed to form flat and shapeless agglomerates, while in the airbrush-spray method, the particles tend to adopt a more spherical shape. Additionally, compared to findings from other researchers [57,58], it is clear that the effect of the coating technique is evident and that developing specialized methods for surface coating could enhance the uniformity of the coating.

#### 3.4.2. EDS Analysis

**Figure 11:**
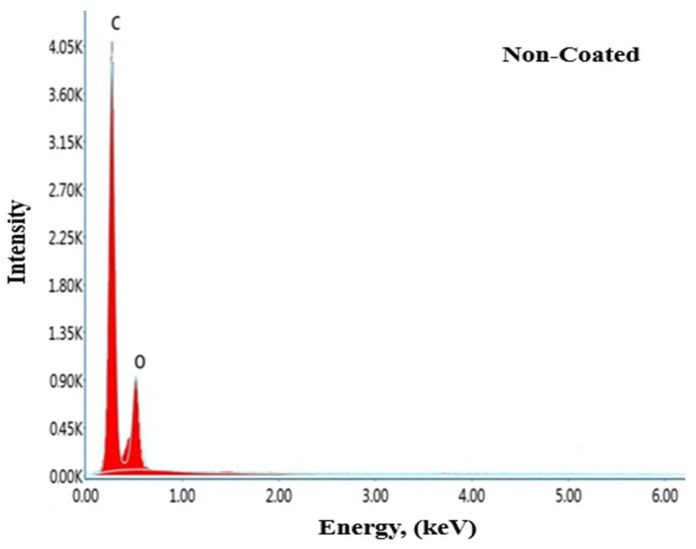
EDS spectrum analyses of the PEEK.

In this study, energy-dispersive X-ray spectroscopy (EDX) analysis was used to determine the chemical composition and elemental distribution of the PEEK material used as a substrate. Figure 11 shows the EDS analysis results of the PEEK material.

When examining the EDS results provided in Figure 11, signals from the oxygen (O) and carbon (C) atoms that make up the PEEK material can be observed. The elemental analysis results obtained from SEM and EDS are consistent with each other.

**Figure 12:**
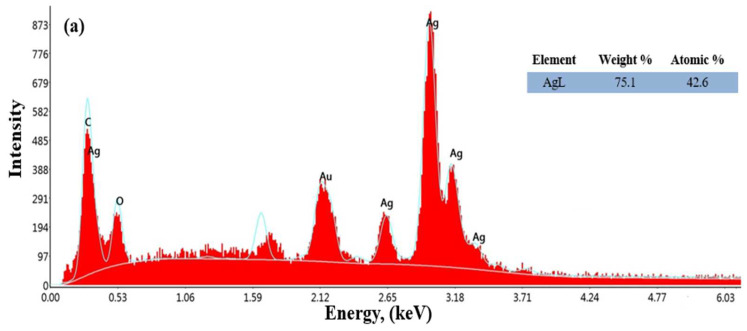

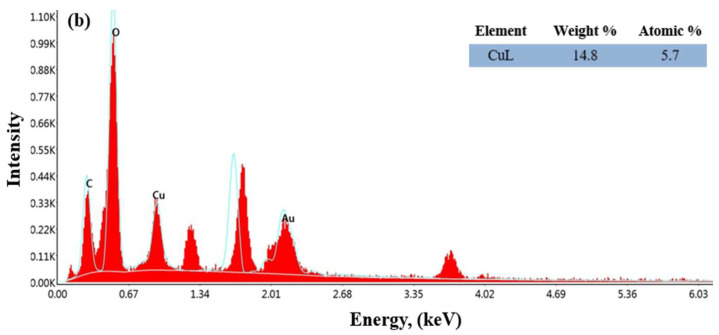
EDS spectrum of AgNPs (**a**) and CuNPs (**b**) synthesized using *E. telmateia* Ehrh. plant extract.

Energy-dispersive X-ray spectroscopy (EDX) analysis was used to determine the chemical composition and elemental distribution of the Ag and Cu nanoparticles obtained through green synthesis and used as coating materials in this study. Figure 12a,b show the EDS spectra for AgNPs and CuNPs, respectively.

According to the results shown in Figure 12a, the elemental composition of AgNPs has been confirmed, with the structure containing approximately 73% Ag. In Figure 12b, the elemental composition of CuNPs shows that they contain about 15% copper, and this composition has been verified. During the SEM-EDS analysis of silver and copper nanoparticles, unidentified elements were detected, which could be attributed to the green synthesis method. These elements likely originated from the biological extracts used in the synthesis process, such as plant materials, which contain various trace elements. Characteristic absorption peaks of CuNPs were observed at 1 keV, while those for AgNPs were observed in the range of 0.5 to 3 keV. As a result, the shape, size, and distribution of the nanoparticles are consistent with studies where Ag and Cu nanoparticles were synthesized using green synthesis methods. Similar results have been reported in the literature for metal nanoparticle synthesis using plant-based green synthesis methods [57,59]. The analyses also show signals from oxygen (O) and carbon (C) atoms along with the other signals. These signals from oxygen (O) and carbon (C) atoms are interpreted to be due to the drying atmosphere in the oven. Gold (Au) atom signals are attributed to the gold coating during the EDS measurement.

**Figure 13:**
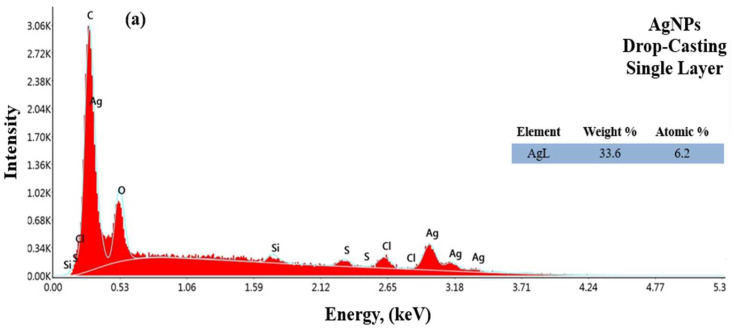

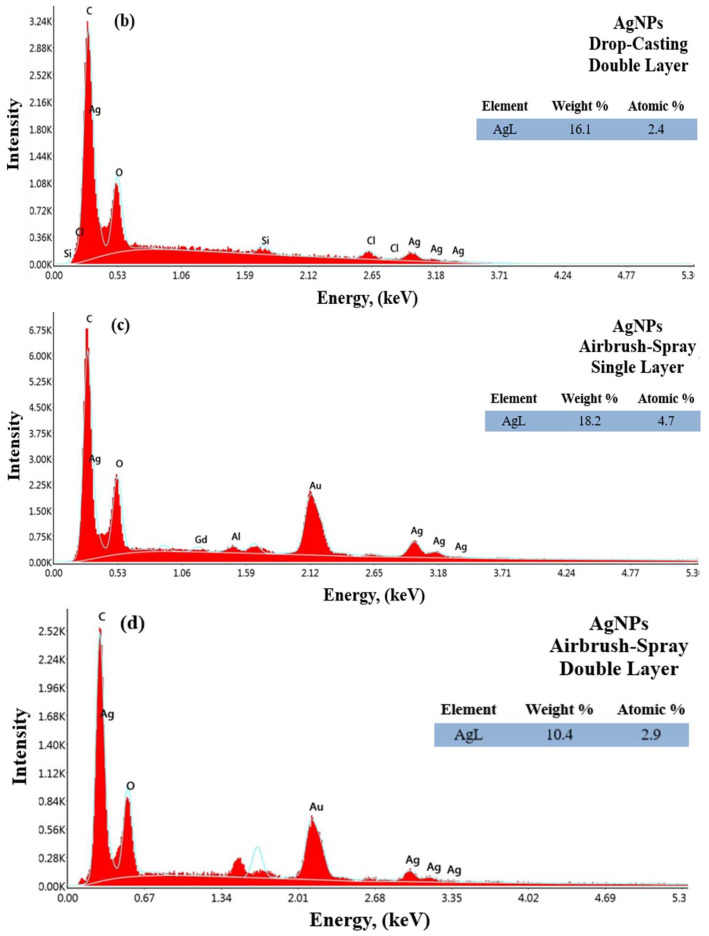
EDS spectrum of PEEK samples coated with AgNPs drop-casting single layer (**a**), double layer (**b**) and airbrush-spray single layer (**c**), double layer (**d**).

In this study, energy-dispersive X-ray spectroscopy (EDX) analysis was used to determine the chemical composition and elemental distribution of the substrate and samples coated with four different parameters. Figure 13a–d show the EDS analysis results for AgNPs with single- and double-layer coatings using the drop-coating and airbrush-spray coating methods, respectively.

**Figure 14:**
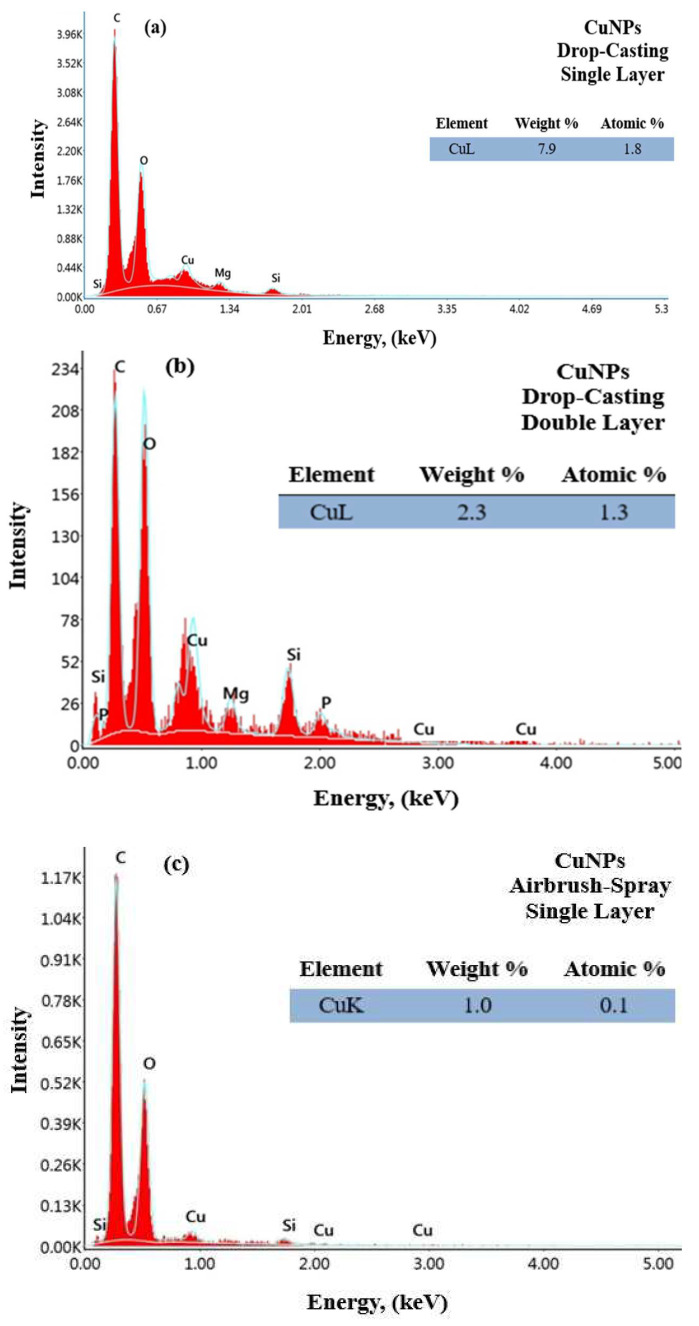

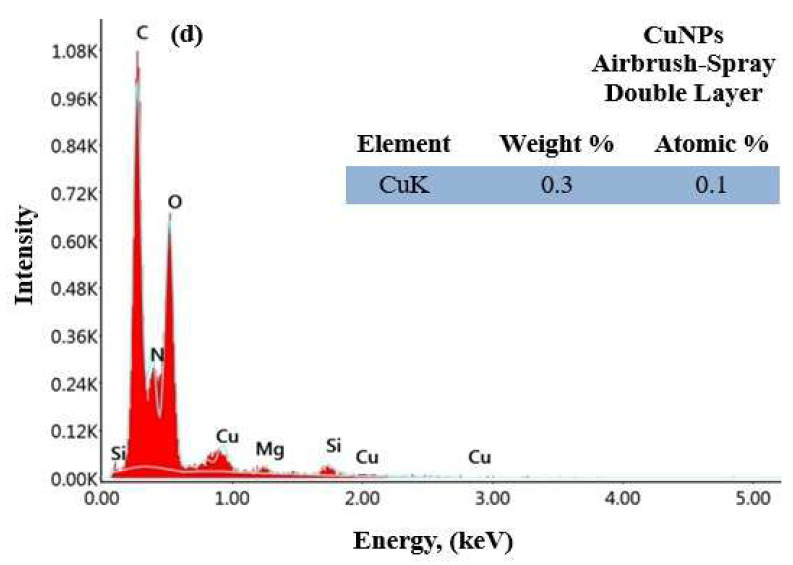
EDS spectrum of PEEK samples coated with CuNPs drop-casting single layer (**a**), double layer (**b**) and airbrush-spray single layer (**c**), double layer (**d**).

According to the results obtained in Figure 13a–d, characteristic absorption peaks of AgNPs in the 0.5 to 3 keV range were observed for both coating methods. As a result, the shape, size, and distribution of the NPs are consistent with studies that synthesized AgNPs using green synthesis methods. The results confirmed the elemental composition of the AgNPs, showing that the structure contains approximately 34% and 16% silver in single and double coatings applied by the drop-casting method, respectively, and approximately 18% and 10% silver in coatings applied by the airbrush-spray method. EDS analyses revealed Ag and Cl elements that confirm the formation of AgNPs and also showed signals from C and O elements, which are thought to originate from the drying atmosphere of the oven. These results are consistent with the literature [60,61]. Figure 14a–d show the EDS analysis results for CuNPs with single- and double-layer coatings using the drop-coating and airbrush-spray-coating methods, respectively.

Based on the results obtained in Figure 14a–d, characteristic absorption peaks for CuNPs were observed at 1 keV for both coating methods. It was observed that the peaks widened and the absorbance values decreased in the airbrush-spray coating compared to the drop-casting coating. As a result, the shape, size, and distribution of the NPs are consistent with studies where CuNPs were synthesized using green synthesis methods. The elemental composition of CuNPs has been confirmed, showing that coatings applied using the drop-casting method contain approximately 8% and 2% copper for single and double coatings, respectively, while those applied using the airbrush-spray method contain approximately 1% and 0.3% copper. EDS analyses also detected peaks for C, N, and O elements, which are thought to originate from the drying atmosphere in the oven. Additionally, signals from Mg, P, and Si elements were observed. These results are consistent with studies in the literature [62,63,64]. The elements and molecules detected beyond the chemical structures of AgNPs, CuNPs, and PEEK are thought to originate from the plant extract used in nanoparticle production and the coating applied during the analysis.

### 3.5. Surface Roughness Results

**Figure 15:**
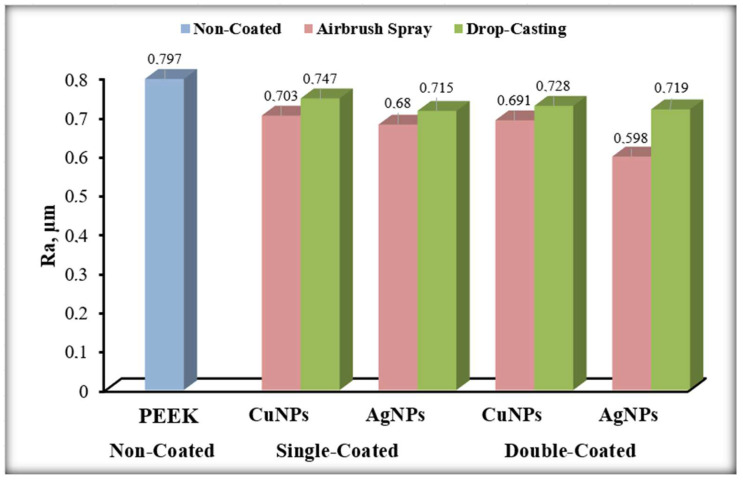
Surface roughness results.

It is very important to measure surface roughness to evaluate the effectiveness of the coating. Surface roughness plays a significant role in the tissue compatibility of implant materials. In this study, surface roughness measurements were carried out to observe the effect of the coating, and the results are presented in Figure 15. Non-coated PEEK was used as the control group and compared with other samples. Measurements were taken from five different points on each sample, and the average of these measurements was used. For some biomaterials, fine and medium surface roughness values range between 0.5 and 1 μm and 1 to 2 μm, respectively [65]. The surface roughness values obtained indicate that the coating techniques show promise for biomedical applications, as they exhibit medium-level surface roughness. Jothi et al. have shown how the surface roughness properties of the coating affect various characteristics such as hydrophobic properties, interactions with bacteria, and resistance to abrasives [66,67]. These properties are enhanced by increased surface roughness. Therefore, the surface roughness values obtained in our study can be considered significant.

When the AgNPs single- and double-layer airbrush-spray coated PEEK samples were examined, a decrease of 0.082 µm in surface roughness was observed as the number of coating layers increased. Based on this result, it can be said that the number of layers is important in coatings made by the airbrush-spray coating method, and the surface roughness decreases as the number of coating layers increases compared to non-coated PEEK. When the AgNPs single- and double-layer drop-casting coated PEEK samples were compared, it was observed that the surface roughness increased by 0.004 µm as the number of coating layers increased. This result indicates that there is no significant difference between the single and double layers of the drop-casting method. Considering that the drop-casting method results in a spread due to the application process, these close results are expected.

In general, when the surface roughness of the AgNPs samples was evaluated for both the airbrush-spray and drop-casting methods, it was observed that the airbrush-spray method reduced the surface roughness more significantly compared to the drop-casting method. The airbrush-spray method resulted in a more homogeneous coating, creating a more stable structure between layers.

When the CuNPs single- and double-layer airbrush-spray coated PEEK samples were examined, a decrease of 0.012 µm in surface roughness was observed as the number of coating layers increased. The airbrush-spray method also reduced surface roughness in the CuNPs coatings. When the CuNPs single- and double-layer drop-casting coated PEEK samples were examined, a decrease of 0.019 µm in surface roughness was observed as the number of coating layers increased. The drop-casting method reduced surface roughness in CuNPs coatings as well. When the results were compared according to the coating methods, it was found that the samples coated by airbrush-spray showed more significant differences in reducing the surface roughness compared to those coated by the drop-casting method. When comparing the single and double layers, in both methods, an approximate 2% decrease in surface roughness was observed as the number of layers increased. The percentage changes in surface roughness are provided in Table 3.

When AgNPs and CuNPs were compared with each other, it was observed that AgNPs-coated samples reducing surface roughness more effectively compared to CuNPs-coated samples. When SEM analyses were examined, it could be said that the most important factor causing this situation was the size difference between AgNPs and CuNPs. It was observed that AgNPs did not agglomerate and this affected the particle sizes and created the surface roughness difference between the two materials.

### 3.6. Release Results

The release studies were monitored for 7 days on PEEK samples coated with silver and copper nanoparticles. As understood from the results obtained from the UV analysis graphs, no release occurred from the single-layer and double-layer coatings applied by airbrush-spray and drop-casting methods over the 7-day period. Since the coatings are of great importance in modifying the PEEK surface, the lack of release from the coatings is a desired outcome. In this way, the surfaces modified with metallic nanoparticles will continue to exhibit the distinct properties of silver and copper nanoparticles separately. It is believed that these distinctive properties acquired by the samples are of great importance in biological studies [68].

### 3.7. Antioxidant Activity Test Results

**Figure 16:**
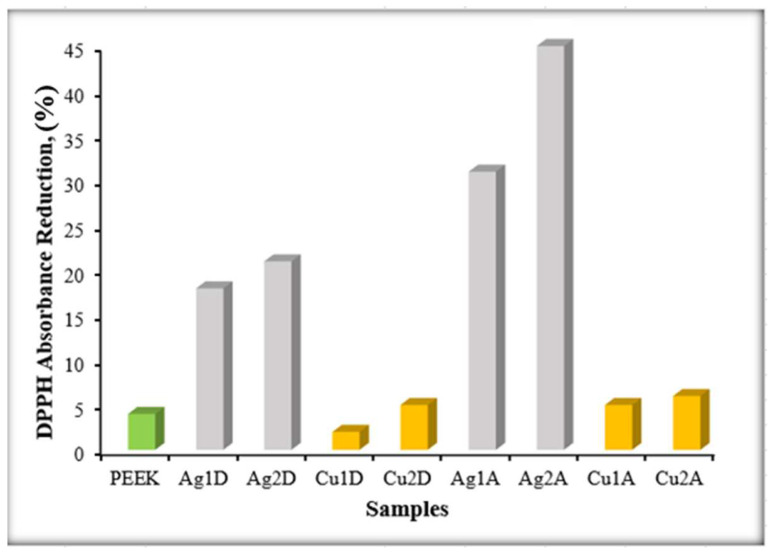
Antioxidant activity results.

The antioxidant activities of non-coated PEEK, AgNPs-coated PEEK, and CuNPs-coated PEEK samples are shown in Figure 16. When comparing non-coated PEEK with AgNPs-coated samples, it was observed that AgNPs/single-layer/drop-casting (Ag1D), AgNPs/double-layer/drop-casting (Ag2D), AgNPs/single-layer/airbrush-spray (Ag1A), and AgNPs/double-layer/airbrush-spray (Ag2A) samples exhibited antioxidant activity and increased the antioxidant activity of PEEK. Accordingly, it was noted that as the coating amount increased, the antioxidant activity also increased, and the antioxidant activity varied depending on the coating method. It can be said that the samples coated using the airbrush-spray method with AgNPs showed higher antioxidant activity compared to those coated using the drop-casting method. The primary reason for this is that the airbrush-spray method provides a more homogeneous distribution.

When comparing non-coated PEEK with CuNPs-coated samples, no significant effect was observed in CuNPs/single-layer/drop-casting (Cu1D), CuNPs/double-layer/drop-casting (Cu2D), CuNPs/single-layer/airbrush-spray (Cu1A), and CuNPs/double-layer/airbrush-spray (Cu2A) samples. Thus, it cannot be said that CuNPs significantly increased the antioxidant activity of PEEK. While the coating amount affected the antioxidant potential in the drop-casting method, the coating amount did not cause a significant change in the airbrush-spray method. When comparing samples coated with CuNPs using the drop-casting and airbrush-spray methods, a difference was observed between Cu1D and Cu1A, while no significant difference was found between Cu2D and Cu2A. In fact, Cu2D and Cu1A exhibited the same antioxidant effect. When comparing AgNPs-coated and CuNPs-coated samples, it was found that AgNPs provided more favorable antioxidant activity compared to CuNPs [69]. The differences in antioxidant activity among the samples can be attributed to several factors. The first is the type of nanoparticles used: AgNPs typically exhibit stronger antioxidant properties than CuNPs due to their superior ability to scavenge free radicals. Additionally, the coating method affects the distribution of nanoparticles, with airbrush-spray leading to a more uniform coating compared to drop-casting. The thickness of the coating also plays a role; increased layers of AgNPs enhance antioxidant activity, as they have a larger surface area for interaction. In contrast, aggregation of CuNPs can diminish their effectiveness. Finally, the intrinsic properties of the nanoparticles, such as size and reactivity, further influence antioxidant performance. Addressing these factors provides a clearer understanding of the variations in antioxidant activity among the coated and non-coated PEEK samples.

### 3.8. Determination of Cell Viability by In Vitro Cytotoxicity Analysis

**Figure 17:**
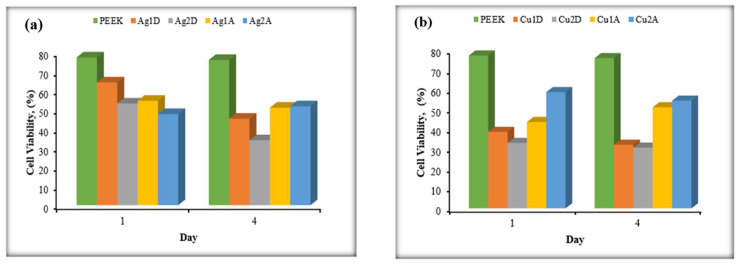
MTT test results of AgNPs (**a**) and CuNPs (**b**).

In vitro cytotoxicity tests were conducted for Ag1D, Ag2D, Ag1A, Ag2A, Cu1D, Cu2D, Cu1A, Cu2A, and non-coated PEEK. Non-coated PEEK was accepted as a reference for other coated samples, and cytotoxicity calculations were made accordingly. The results for AgNP and CuNP are shown in Figure 17a,b, respectively.

Samples from both coated and non-coated PEEK were taken on days 1 and 4. Based on the MTT values of the collected samples, it was observed that AgNPs reduced cell viability up to day 4. While silver is known to aid in wound healing, its concentration plays a crucial role in determining its effect on cell viability. According to the results obtained, there was also a difference in toxic effects between the 1-layer and 2-layer coatings. When evaluating the cytotoxicity results of CuNPs, it was found that they exhibited cytotoxicity on all sample days, indicating that special care should be taken to avoid direct contact between copper nanoparticles and human tissues when considering their potential use in clinical settings [70]. Contrary to expectations, it was observed that the one-layer coating of CuNPs exhibited a more toxic effect than the two-layer coating. Considering that CuNPs tend to agglomerate, it can be said that the toxic effect in the two-layer coating is reduced due to the smaller surface area in contact with the cells.

During the comparison of coating methods, it was observed that the airbrush-spray method exhibited a more toxic effect than the drop-casting method. The reason for this is that the airbrush-spray method allows for a more homogeneous distribution, resulting in a larger surface area in contact with the cells compared to the drop-casting method. When AgNPs and CuNPs are compared, CuNPs were found to exhibit more toxic effects than AgNPs. This highlights the importance of not only the metallic nanoparticle type but also the method of application in determining toxicity. Additionally, these findings suggest that toxicity and biocompatibility are not solely dictated by nanoparticle composition but also by how these particles interact with biological systems at a cellular level. In this context, the relationship between antioxidant activity and biocompatibility is complex. A compound with high antioxidant activity does not automatically ensure biocompatibility, as factors like immune response, metabolism, and dosage must also be taken into account when assessing biocompatibility [71].

### 3.9. Antibacterial Activity Test Results

**Figure 18:**
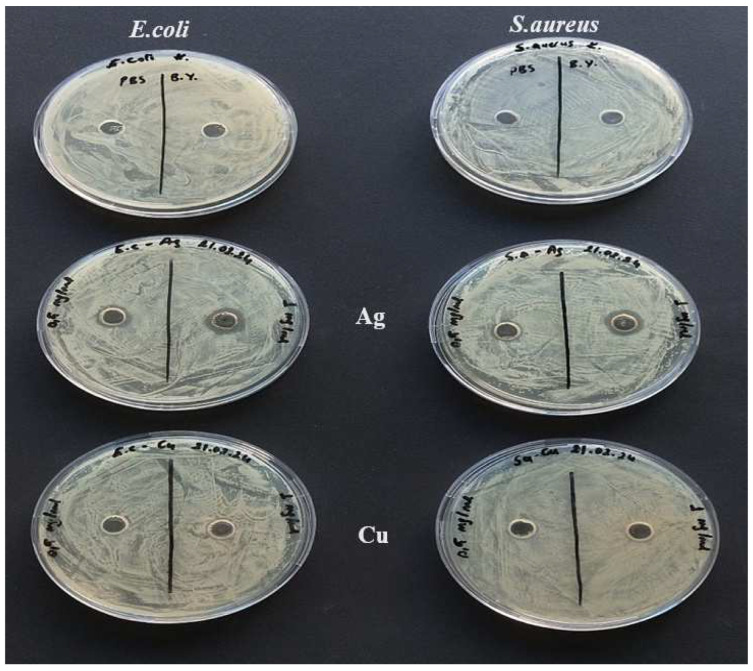
Antibacterial activities of AgNPs and CuNPs.

The activities of AgNPs and CuNPs solutions against Gram-negative *E. coli* and Gram-positive *S. aureus* bacteria were investigated. It was observed that AgNPs exhibited antibacterial activity against both types of bacteria, while CuNPs were not as effective as AgNPs. The antibacterial activities of AgNPs and CuNPs are shown in Figure 18. By measuring the zone diameters, it was found that the 1.0 mg/mL AgNPs solution exhibited greater antibacterial activity compared to the 0.5 mg/mL AgNPs solution. It can be stated that as the silver content in the solution increases, antibacterial activity also increases. Accordingly, regardless of the coating method, an increase in the number of AgNPs coating layers enhances the antibacterial activity. When comparing the effectiveness against Gram-positive and Gram-negative bacteria, it is thought that the lower effectiveness in *S. aureus* compared to *E. coli* is due to differences in their cell walls [72].

## 4. Conclusions

In this study, the surface properties, antioxidant activity, and antibacterial effects of PEEK substrates coated with green-synthesized silver and copper nanoparticles were evaluated. The coatings enhanced the surface quality of PEEK, with AgNPs showing superior antibacterial and antioxidant properties compared to CuNPs. The use of airbrush-spray and drop-casting methods for coating yielded satisfactory results in terms of nanoparticle dispersion, coating thickness, and surface roughness. The results suggest that PEEK materials coated with AgNPs, in particular, hold significant promise for biomedical applications due to their enhanced bioactivity and surface quality. Future studies could explore the in vivo effects and long-term durability of these coatings for wider applications.

## Figures and Tables

**Table 2:** Experimental coating details.

Code	Material	Coating Method	Coating Layer
PEEK	Polyetheretherketone	Non-Coated	Non-Coated
Ag1D	Silver (Ag)	Drop-Casting	Single Layer
Ag2D			Double Layer
Ag1A		Airbrush-Spray	Single Layer
Ag2A			Double Layer
Cu1D	Copper (Cu)	Drop-Casting	Single Layer
Cu2D			Double Layer
Cu1A		Airbrush-Spray	Single Layer
Cu2A			Double Layer

**Table 3:** Surface roughness percentage differences.

Percentages Compared to Non-Coated Samples				
Coating Method	Single-Coated		Double-Coated	
	AgNPs	CuNPs	AgNPs	CuNPs
Airbrush-Spray	−17%	−13%	−33%	−15%
Drop-Casting	−11%	−7%	−11%	−9%

## Data Availability

The original contributions presented in the study are included in the article, and further inquiries can be directed to the corresponding authors.
